# An Improved Scalable Synthesis of α- and β-Amyrin

**DOI:** 10.3390/molecules23071552

**Published:** 2018-06-27

**Authors:** Immo Serbian, René Csuk

**Affiliations:** Martin-Luther-University Halle-Wittenberg, Organic Chemistry, Kurt-Mothes-Str. 2, D-06120 Halle (Saale), Germany; immo.serbian@chemie.uni-halle.de

**Keywords:** α-amyrin, β-amyrin, partial synthesis

## Abstract

The synthesis of α- and β-amyrin was accomplished starting from easily accessible starting materials, oleanolic, and ursolic acid. The procedures allow the preparation of β-amyrin in an exceptionally short scalable manner via selective iodation and reduction. For α-amyrin, a different synthetic approach had to be chosen providing access to α-amyrin in medium-to-large scale.

## 1. Introduction

**Figure 1:**
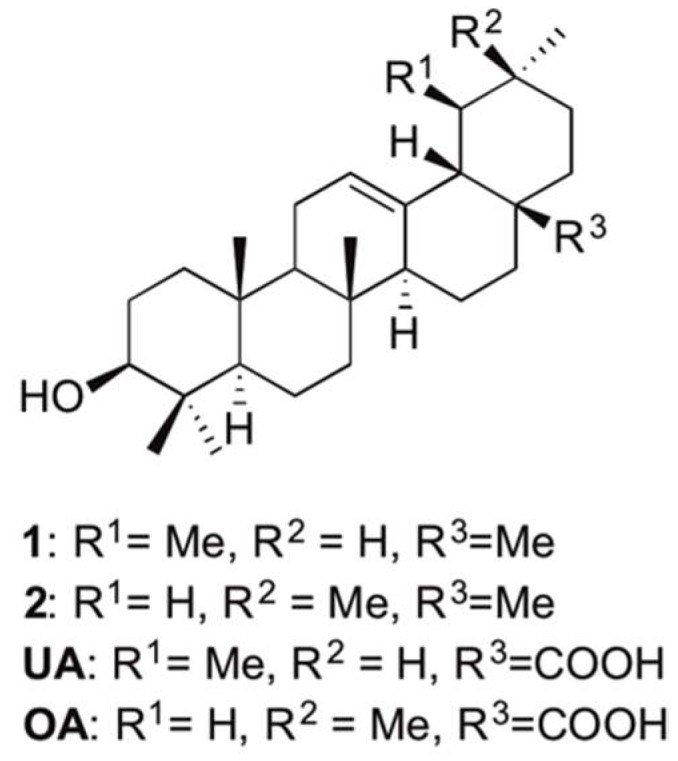
Basic structure of pentacyclic triterpenes of the amyrin family.

Pentacyclic triterpenoids of the amyrin family (Figure 1) represent a class of the most abundant secondary metabolites in plants sharing plant protective properties [1,2]. Among their high value as antimicrobial [3] and antifungal [4] agents in plants, they share pharmacologically relevant properties, and have proven anti-inflammatory [5,6,7], anti-nociceptive [8], and gastro-protective [9] activities, and inhibit HIV transcriptase-1 [10]. In addition, studies show an increasing biological activity by derivatization. As a consequence, this compound class still receives interest, and offers possible candidates for enhanced bioactivity [11].

In spite of belonging to the most abundant secondary metabolites, the problem for accessing larger quantities of α-amyrin (**1**) and β-amyrin (**2**) persists [12]. Amyrins represent a precursor for more complex triterpenoids like boswellic acid (found in frankincense) or maslinic acid (found in olives), but their concentration in plant tissue is rather low compared to other triterpenoids, such as oleanolic acid (**OA**) and ursolic acid (**UA**). Furthermore, their purification is challenging, especially in large scale. Over the past decades, there have been several studies on purification and identification of sources for these compounds. However, it is still an ongoing task to gain access to α- and β-amyrin [6,13]. The first partial synthesis of β-amyrin was done by Barton in 1968 [14], and the first total synthesis of β-amyrin was accomplished by E.J. Corey in 1993 [15]. Different synthetic approaches also targeted the β-amyrin and triterpenoids of the β-amyrin family [16,17,18]. Until now, there has been no publication describing the total synthesis of α-amyrin. A recent partial synthesis, starting from easily accessible starting materials—oleanolic acid (**OA**) and ursolic acid (**UA**)—made α-amyrin, β-amyrin, and lupeol accessible [19].

Our aim was to improve the synthetic route to gain access to larger quantities of α- and β-amyrin to enable further derivatization and research.

## 2. Results and Discussion

### 2.1. Synthesis

#### 2.1.1. Unsuccessful Approaches

The first approaches of a direct reduction of oleanolic and ursolic acid by reaction with tris(pentafluorophenyl)borane and alkylsilanes did not afford the desired reaction product [20,21]. Likewise, an Arndt–Eistert reaction followed by decarboxylation was not successful, because we were not able to obtain the desired products.

#### 2.1.2. High Yield Preparation of β-Amyrin

**Figure 2:**
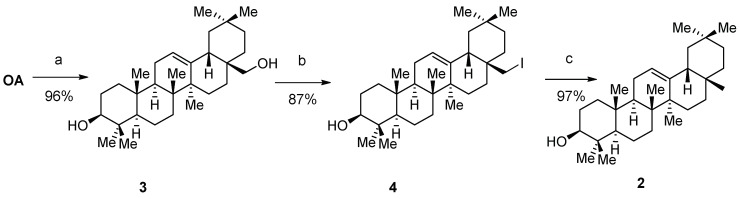
(**a**) LiAlH_4_, THF, reflux, 12 h; (**b**) I_2_, imidazole, PPh_3_, THF, reflux, 10 min; (**c**) Zn, AcOH, r.t. 4 h.

A straightforward approach, previously already used in the reduction of decahydronapthalenes, led to good results (Figure 2) [22]. Thus, starting from oleanolic acid (**OA**) its reduction with lithium aluminum hydride afforded the corresponding alcohol **3** in high yield. Therefore, the next step was carried out without any further purification. In an Appel-like reaction, a selective substitution of the primary hydroxy group with iodine proceeded in short reaction times and good isolated yields. Longer reaction times, however, led to the formation of unwanted byproducts. After purification of compound **4**, a reduction with zinc powder gave β-amyrin, and again, no further purification was necessary. Therefore, over three steps, we were able to convert oleanolic acid to β-amyrin in an 81% overall yield. This represents a good improvement to the previously reported 42% [19]. Unfortunately, this approach did not work with ursolic acid; due to its limited reactivity on C-28, the substitution reaction with iodine at C-28 did not proceed well.

#### 2.1.3. Preparation of α- and β-Amyrin

**Figure 3:**
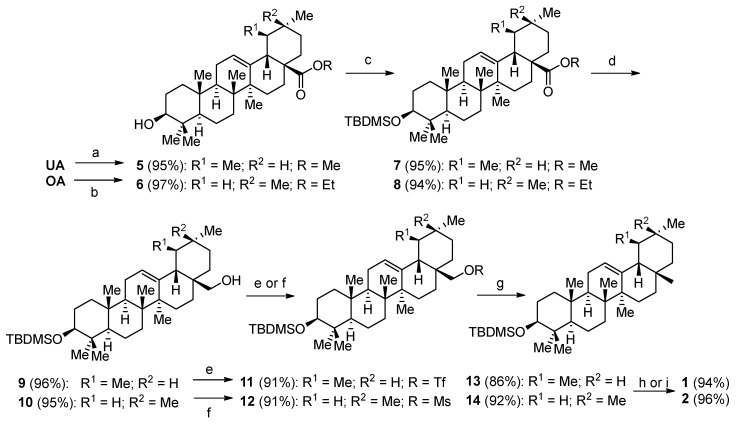
(**a**) K_2_CO_3_, MeI, DMF, 25 °C, 12 h; (**b**) K_2_CO_3_, EtI, DMF, r.t. 10 h; (**c**) TBDMSCl, imidazole, DMF, 50 °C, 24 h; (**d**) LiAlH_4_, THF, 3 h; (**e**) MsCl, NEt_3_, DCM, r.t. 1 h; (**f**) Tf_2_O, py, DCM, 0 °C; (**g**) LiAlH_4_, THF, 2 h; (**h**) I_2_, MeOH, reflux, 3 h; (**i**) Bu_4_NF, THF, reflux, 8 h.

Therefore, we decided to introduce a different leaving group than iodine. Unfortunately, a protection of the hydroxy group at C-3 was necessary (Figure 3). For oleanolic acid methansulfonic esters (**12**) represented a suitable leaving group and resulted in good yields in the reduction to follow, to yield the corresponding alkane **14**. However, it was not possible to carry out the same protocol with ursolic acid as a starting material. It was already known that the shift of a methyl group in the α-amyrin type triterpenoids reduces the reactivity at the carboxylic group of ursolic acid compared to the β-amyrin type oleanolic acid. This holds true for the corresponding alcohol **9** as well. It was not possible to reduce the mesylate with lithium aluminum hydride to alkane **13**. Different leaving groups, such as iodine or tosylates, could not be introduced. With trifluoromethanesulfonic anhydride, however, it was possible to generate triflate **11**. This triflate (stable at −20 °C for a couple of days in the dark) could be reduced with lithium aluminum hydride to yield the TBDMS-protected α-amyrin-ether **13**. This approach yielded α-amyrin in 64% overall yield, while β-amyrin was formed in around 69% overall yield. The latter yield is a significant improvement to known procedures for the synthesis of β-amyrin [19]. Upon scaling up of the reactions by a factor of 5, the overall yield drops slightly (62 and 66%, respectively).

## 3. Experimental

### α-Amyrin *(**1**)*

Procedure 1: Compound **13** (300 mg, 0.55 mmol) was dissolved in 1 m tetrabutylammonium fluoride in THF (10 mL). After stirring for 14 h at room temperature, the reaction mixture was diluted with diethyl ether (20 mL) and washed with water (10 mL) and brine (10 mL). The organic phase was dried over magnesium sulfate, filtrated, and the solvent was removed under reduced pressure. The crude product was purified by column chromatography (silica gel, hexane/EtOAc, 9:1) to yield **1** as a colorless crystalline solid (220 mg, 94%).

Procedure 2: Compound **13** (100 mg, 0.18 mmol) was dissolved in methanol (50 mL), and iodine (23 mg, 0.18 mmol) was added. The reaction mixture was heated under reflux for 3 h. After cooling to room temperature, the reaction was quenched by adding a solution of sodium thiosulfate and water. The aqueous solution was extracted with diethyl ether (3 × 20 mL), and the combined organic extracts were washed with brine and dried over magnesium sulfate. Purification by column chromatography (silica gel, hexane/EtOAc, 9:1) gave **1** as a colorless crystalline solid (53 mg, 69%). m.p. = 178–181 °C (lit.: 180–181 °C) [23]; ${\left[\alpha \right]}_{D}^{20}$ = +84.8° (*c* 0.315, CHCl_3_) (lit.: +83.5° (*c* 1.000, CHCl_3_)) [24]; R*_f_* = 0.24 (silica gel, hexane/EtOAc, 9:1); IR (KBr): 3442*br*, 2947*s*, 2931*s*, 2873*m*, 2853*m*, 1637*w*, 1458*m*, 1378*m*, 1081w, 1036*m*, 996*s*, 666*m* cm^−1^; ^1^H NMR (500 MHz, CDCl_3_): δ = 5.13 (*dd*, *J* = 3.6 Hz, 3.6 Hz, 1H, H-12), 3.22 (*dd*, *J* = 11.0, 5.2 Hz, 1H, H-3), 2.05–1.95 (*m*, 1H, H-16a), 1.95–1.89 (*m*, 2H, H-11a + H-11b), 1.88–1.79 (*m*, 1H, H-15a), 1.70–1.58 (*m*, 3H, H-1a + H-2a + H-2b), 1.58–1.49 (*m*, 3H, H-7a + H-6a + H-9), 1.46–1.18 (*m*, 8H, H-22a + H-6b + H-21a + H-7b + H-19 + H-18 + H-22b + H-21b), 1.07 (*s*, 3H, H-27), 1.06–0.95 (*m*, 2H, H-1b + H-15b), 1.01 (*s*, 3H, H-23), 1.00 (*s*, 3H, H-24), 0.96 (*s*, 3H, H-25), 0.86 (*d*, *J* = 5.5 Hz, 3H, H-30), 0.89–0.83 (*m*, 2H, H-16b + H-20), 0.80 (*s*, 3H, H-28), 0.79 (*s*, 3H, H-26), 0.79 (*d*, *J* = 6.1 Hz, 3H, H-29), 0.76–0.71 (*m*, 1H, H-5) ppm; ^13^C NMR (125 MHz, CDCl_3_): δ = 139.7 (C-13), 124.6 (C-12), 79.2 (C-3), 59.2 (C-18), 55.4 (C-5), 47.9 (C-9), 42.3 (C-14), 41.7 (C-22), 40.2 (C-8), 39.8 (C-19), 39.8 (C-20), 39.0 (C-1), 38.9 (C-10), 37.1 (C-4), 33.9 (C-17), 33.1 (C-7), 31.4 (C-21), 28.9 (C-28), 28.3 (C-24), 28.3 (C-16), 27.5 (C-2), 26.8 (C-15), 23.5 (C-11), 23.4 (C-27), 21.6 (C-30), 18.5 (C-6), 17.6 (C-29), 17.0 (C-23), 15.8 (C-25), 15.8 (C-26) ppm; MS (ASAP): *m*/*z* (%) = 409.2 ([M-H_2_O + H]^+^, 100), 427.2 ([M − H]^+^, 16).

### β-Amyrin; (3β) Olean-12-en-3-ol *(**2**)*

Procedure 1: Compound **14** (300 mg, 0.55 mmol) was dissolved in 1 m tetrabutylammonium fluoride in THF (10 mL). After 14 h of stirring at room temperature, the reaction mixture was diluted with diethyl ether (20 mL) and washed with water (10 mL) and brine (10 mL). The organic phase was dried over magnesium sulfate, filtrated, and the solvent was removed under reduced pressure. The crude product was purified by column chromatography (silica gel, hexane/EtOAc, 8:2) to yield **2** as a colorless crystalline solid (225 mg, 96%).

Procedure 2: Compound **4** (4 g, 7.24 mmol) was dissolved in acetic acid (1 L) at 40 °C. Zinc (2.5 g, 38.2 mmol) was added, and the reaction was stirred for 4 h at room temperature. The zinc was filtered off, and the filtrate was concentrated under reduced pressure at 40 °C. As soon as β-amyrin began to crystalize, the product was precipitated by adding water (1 L). The precipitate was filtered off and washed thoroughly with water. The white crystalline solid was dried to furnish **2** as a white crystalline solid (2.99 g, 97%). m.p. = 195–197 °C (lit.: 197–198 °C) [25]; ${\left[\alpha \right]}_{D}^{20}$ = +89° (*c* 0.315, CHCl_3_) (lit.: +89° (*c* 0.5, CHCl_3_)) [26]; R*_f_* = 0.33 (silica gel, hexane/EtOAc, 8:2); IR (KBr): ν = 3430*br*, 2968*s*, 2946*s*, 2926*s*, 2872*m*, 2854*m*, 1632*m*, 1464*m*, 1384*m*, 1362*w*, 1096*w*, 1078*w*, 1032*w*, 996*w* cm^−1^; ^1^H NMR (500 MHz, CDCl_3_): δ = 5.18 (*dd*, *J* = 3.6, 3.6 Hz, 1H, H-12), 3.26–3.17 (*m*, 1H, H-3), 2.03–1.81 (m, 4H, H-16a + H-18 + H-11a + H-11b), 1.81–1.72 (*m*, 1H, H-15a), 1.72–1.47 (*m*, 7H, H-19a + H-1a + H-2a + H-2b + H-9 + H-6a + H-7a), 1.47–1.38 (*m*, 2H, H-22a + H-6b), 1.38–1.28 (*m*, 2H, H-21a + H-7b), 1.28–1.15 (*m*, 1H, H-22b), 1.13 (*s*, 3H, H-27), 1.15–0.91 (*m*, 4H, H-21b + H-15b + H-2b + H-19b), 1.00 (*s*, 3H, H-24), 0.97 (*s*, 3H, H-26), 0.94 (*s*, 3H, H-25), 0.87 (*s*, 6H, H-30 + H-29), 0.90–0.76 (*m*, 1H, H-16b), 0.83 (*s*, 3H, H-28), 0.79 (*s*, 3H, H-23), 0.74–0.70 (*m*, 1H, H-5) ppm; ^13^C NMR (125 MHz, CDCl_3_): δ = 145.3 (C-13), 121.9 (C-12), 79.2 (C-3), 55.4 (C-5), 47.8 (C-9), 47.4 (C-18), 47.0 (C-19), 41.9 (C-14), 40.0 (C-8), 38.9 (C-4), 38.8 (C-1), 37.3 (C-22), 37.1 (C-10), 34.9 (C-21), 33.5 (C-29), 32.8 (C-17), 32.7 (C-7), 31.2 (C-20), 28.6 (C-28), 28.3 (C-24), 27.4 (C-2), 27.1 (C-16), 26.3 (C-15), 26.2 (C-27), 23.9 (C-30), 23.7 (C-11), 18.6 (C-6), 17.0 (C-26), 15.8 (C-25), 15.7 (C-23) ppm; MS (ASAP): *m*/*z* (%) = 409.6 ([M − H_2_O + H]^+^, 100), 427.6 ([M + H]^+^, 22).

### (3β) Olean-12-en-3,28-diol, (+)-erythrodiol *(**3**)*

Lithium aluminum hydride (1.17 g, 30.6 mmol) was dissolved in THF (100 mL), and a solution of **OA** (2.00 g, 4,379 mmol) in THF (70 mL) was added slowly at 0 °C. The reaction was heated under reflux until TLC showed the complete consumption of **OA**. Surplus lithium aluminum hydride was quenched by adding THF/water (1:1) at 0 °C. The mixture was acidified with 2 M hydrochloric acid and extracted with diethyl ether (3 × 50 mL). The combined organic phases were washed with brine and dried over magnesium sulfate. The solvent was removed to afford **3** as a colorless crystalline solid (1.86 g, 96%). m.p. = 226–228 °C (lit.: 225–229 °C) [27]; ${\left[\alpha \right]}_{D}^{20}$ = +75° (*c* 0.325, CHCl_3_) (lit.: +70°) [27]; R*_f_* = 0.24 (silica gel, chloroform/hexane/EtOAc, 10:8:2); IR (KBr): ν = 3444*br*, 2946*w*, 2866*w*, 1636*m*, 1460*w*, 1384*w*, 1044*w*, 1004*w* cm^−1^; ^1^H NMR (500MHz, CDCl_3_): δ = 5.19 (*dd*, *J* = 3.6, 3.6 Hz, 1H, H-12), 3.55 (*d*, *J* = 11.0 Hz, 1H, H-28a), 3.21 (*d*, *J* = 11.0 Hz, 1H, H-28b), 3.28–3.15 (*m*, 1H, H-3), 1.98 (*dd*, *J* = 13.5, 4.6 Hz, 1H, H-18), 1.94–1.79 (*m*, 3H, H-16a + H-2a + H-2b), 1.77–1.66 (*m*, 2H, H-15a + H-19a), 1.65–1.46 (*m*, 7H, H-1a + H-11a + H-11b + H-6a + H-9 + H-22a + H-7a), 1.46–1.37 (*m*, 1H, H-6b), 1.37–1.25 (*m*, 3H, H-22b + H-7b + H-21a), 1.22–1.13 (*m*, 2H, H-16b + H-21b), 1.16 (s, 3H, H-27), 1.10–1.03 (*m*, 1H, H-19b), 1.03–0.91 (*m*, 2H, H-15b + H-1b), 0.99 (*s*, 3H, H-23), 0.94 (*s*, 3H, H-26), 0.93 (*s*, 3H, H-24), 0.88 (*s*, 3H, H-29), 0.87 (*s*, 3H, H-30), 0.79 (*s*, 3H, H-25), 0.75–0.71 (*m*, 1H, H-5) ppm; ^13^C NMR (125 MHz, CDCl_3_): δ = 144.4 (C-13), 122.5 (C-12), 79.2 (C-3), 69.8 (C-28), 55.3 (C-5), 47.7 (C-9), 46.6 (C-19), 42.5 (C-18), 41.9 (C-14), 39.9 (C-8), 38.9 (C-4), 38.8 (C-1), 37.1 (C-10), 34.3 (C-21), 33.3 (C-29), 32.7 (C-7), 31.2 (C-22), 31.2 (C-20), 31.1 (C-17), 28.3 (C-23), 27.4 (C-11), 26.1 (C-27), 25.7 (C-15), 23.7 (C-2), 23.7 (C-30), 22.2 (C-16), 18.5 (C-6), 16.9 (C-26), 15.7 (C-24), 15.7 (C-25) ppm; MS (ASAP): *m*/*z* (%) = 425.6 ([M-H_2_O + H]^+^, 100), 443.6 ([M + H]^+^, 48).

### (3β) Olean-12-en-28-iodo-3-ol *(**4**)*

Compound **3** (4.0 g, 9.0 mmol) was dissolved in THF (50 mL) and heated under reflux. Imidazole (2.4 g, 35.3 mmol), triphenylphosphane (4.8 g, 18.3 mmol) and iodine (2.0 g, 15.8 mmol) were added one after another. The reaction was heated for 10 min, cooled, and excess iodine was quenched by adding a saturated sodium thiosulfate solution. The resulting mixture was extracted with diethyl ether (3 × 100 mL), the combined organic phases were washed with brine and dried over magnesium sulfate, and the solvent was removed under reduced pressure. Purification by column chromatography (silica gel, chloroform/hexane/EtOAc, 10:8:2) gave **4** as a colorless crystalline solid (4.3 g, 87%). m.p. = 185–188 °C (decomp); ${\left[\alpha \right]}_{D}^{20}$ = +66° (*c* 0.335, CHCl_3_); R*_f_* = 0.31 (silica gel, hexane/EtOAc, 8:2); IR (KBr): ν = 3442*br*, 2948*s*, 2926*m*, 2864*m*, 1636*w*, 1462*w*, 1384*w*, 1364*w*, 1180*w*, 1096*w*, 1044*w*, 1030*w*, 996*w*, 604*w* cm^−1^; ^1^H NMR (500 MHz, CDCl_3_): δ = 5.24 (*dd*, *J* = 3.6, 3.6 Hz, 1H, H-12), 3.35 (*d*, *J* = 9.9 Hz, 1H, H-28a), 3.25–3.18 (*m*, 1H, H-3), 3.04 (*d*, *J* = 9.9, 1H, H-28b), 2.15 (*dd*, *J* = 13.5, 3.7 Hz, 1H, H-18), 1.96 (*m*, 1H, H-16a), 1.92–1.80 (*m*, 2H, H-11a + H-11b), 1.73 (*m*, 1H, H-19a), 1.65–1.23 (*m*, 12H, H-1a + H-9 + H-15a + H-15b + H-2a + H-6a + H-7a + H-6b + H-21a + H-7b + H-22a + H-22b), 1.16 (*s*, 3H, H-27), 1.21–1.12 (*m*, 2H, H-21b + H-16b), 1.09 (*m*, 1H, H-19b), 1.01–0.91 (*m*, 2H, H-2b + H-1b), 1.00 (*s*, 3H, H-24), 0.93 (*s*, 3H, H-26), 0.93 (*s*, 3H, H-25), 0.90 (*s*, 3H, H-29), 0.87 (*s*, 3H, H-30), 0.79 (*s*, 3H, H-23), 0.75–0.71 (*m*, 1H, H-5) ppm; ^13^C NMR (125 MHz, CDCl_3_): δ = 143.6 (C-13), 123.3 (C-12), 79.1 (C-3), 55.3 (C-5), 47.7 (C-9), 47.6 (C-19), 45.0 (C-18), 41.7 (C-14), 39.9 (C-8), 38.9 (C-4), 38.8 (C-1), 37.1 (C-10), 35.2 (C-17), 34.7 (C-21), 34.7 (C-22), 33.0 (C-29), 32.5 (C-7), 31.3 (C-20), 28.2 (C-24), 27.4 (C-15), 26.3 (C-27), 26.1 (C-2), 25.7 (C-28), 24.6 (C-16), 23.7 (C-11), 23.7 (C-30), 18.5 (C-6), 16.5 (C-26), 15.7 (C-25), 15.7 (C-23) ppm; MS (ASAP): *m*/*z* (%) = 535.6 ([M-H_2_O + H]^+^, 100), 553.6 ([M + H]^+^, 18).

### (3β) Methyl 3-hydroxyurs-12-en-28-oate *(**5**)*

Ursolic acid (20.0 g, 0.044 mol) was dissolved in DMF (250 mL) and potassium carbonate (30.0 g, 0.220 mol) was added. The mixture was stirred for 30 min, and methyl iodide (5 mL, 0.080 mol) was added. After stirring for 12 h at room temperature, the product was precipitated from the mixture by adding water (1 L). The solid was filtered off and washed with 2 M hydrochloric acid (2 × 20 mL), water (2 × 20 mL), and the resulting product was dried in a desiccator yielding **5** as a colorless crystal solid (19.7 g, 95%). m.p. = 172–174 °C (lit.: 172–173 °C) [28]; ${\left[\alpha \right]}_{D}^{20}$ = +62.3° (*c* 0.385, CHCl_3_) (lit.: +49.8° (*c* 1.000, CHCl_3_)) [28]; R*_f_* = 0.25 (silica gel, hexane/EtOAc 8:2); IR (KBr): ν = 3446*br*, 2946*m*, 2926*m*, 2870*w*, 1724*w*, 1636*m*, 1456*w*, 1384*w*, 1232*w*, 1200*w*, 1166*w*, 1144*w*, 1112*w*, 1092*w*, 1044*m*, 1032*m*, 756*m* cm^−1^; ^1^H NMR (500 MHz, CHCl_3_) δ = 5.24 (*dd*, *J* = 3.6, 3.6 Hz, 1H, H-12), 3.60 (*s*, 3H, H-31), 3.21 (*dd*, *J* = 11.1, 4.9 Hz, 1H, H-3), 2.22 (*d*, *J* = 11.0 Hz, 1H, H-18), 2.03–1.95 (*m*, 1H, H-16a), 1.90 (*dd*, *J* = 8.9, 3.6 Hz, 2H, H-11a + H-11b), 1.99 (*td*, *J* = 13.4, 4.5 Hz, 1H, H-2a), 1.70–1.56 (*m*, 7H, H-16b + H-22a + H-1a + H-1b + H-15a + H-22b + H-15b), 1.57–1.41 (*m*, 4H, H-6a + H-9 + H-21a + H-7a), 1.40–1.23 (*m*, 4H, H-6b + H-20 + H-21b + H-7b), 1.07 (*s*, 3H, H-27), 1.06–0.98 (*m*, 2H, H-2b + H-19), 0.98 (*s*, 3H, H-24), 0.93 (*d*, *J* = 6.1 Hz, 3H, H-30), 0.91 (*s*, 3H, H-25), 0.85 (*d*, *J* = 6.5 Hz, 3H, H-29), 0.77 (*s*, 3H, H-23), 0.74 (*s*, 3H, H-26), 0.73–0.69 (*m*, 1H, H-5 ppm; ^13^C NMR (125 MHz, CDCl_3_) δ = 178.2 (C-28), 138.3 (C-13), 125.7 (C-12), 79.2 (C-3), 55.4 (C- 5), 53.0 (C-18), 51.6 (C-31), 48.2 (C-17), 47.7 (C-9), 42.1 (C-14), 39.6 (C-8), 39.2 (C-20), 39.0 (C-19), 38.9 (C-1), 38.8 (C-4), 37.1 (C-10), 36.8 (C-22), 33.1 (C-7), 30.8 (C-21), 28.3 (C-24), 28.2 (C-2), 27.4 (C-15), 24.4 (C-16), 23.8 (C-27), 23.5 (C-11), 21.3 (C-30), 18.5 (C-6), 17.2 (C-29), 17.1 (C-26), 15.8 (C-23), 15.6 (C-25) ppm; MS (ASAP): *m*/*z* (%) = 453.6 ([M − H_2_O + H]^+^, 100), 471.7 ([M + H]^+^, 86).

### (3β) Ethyl 3-hydroxyolean-12-en-28-oate *(**6**)*

Oleanolic acid (10.0 g, 0.022 mol) was dissolved in DMF (200 mL) and potassium carbonate (15.0 g, 0.110 mol) was added. The mixture was stirred for 30 min, and ethyl iodide (3.5 mL, 0.044 mol) was added. After stirring for 12 h at room temperature, the product was precipitated by adding water (800 mL). The product was filtered off and washed with 2 M hydrochloric acid (2 × 20 mL), water (2 × 20 mL); drying in a desiccator gave **6** as a colorless crystalline solid (10.3 g, 97%). m.p. = 175–178 °C (Lit.: 217.5–218.0 °C) [29]; ${\left[\alpha \right]}_{D}^{20}$ = +73.4° (*c* 0.34, CHCl_3_) (lit.: +78.9°) [29]; R*_f_* = 0.68 (silica gel, hexane/EtOAc, 8:2); IR (KBr): ν= 3446*br*, 2946*m*, 2868*w*, 1722*m*, 1636*w*, 1462*w*, 1386*w*, 1364*w*, 1260*w*, 1178*m*, 1162*m*, 1124*w*, 1094*w*, 1064*w*, 1036*m* cm^−1^; ^1^H NMR (500 MHz, CDCl_3_): δ = 5.28 (*dd*, *J* = 3.6, 3.6 Hz, 1H, H-12), 4.08 (*qq*, *J* = 10.8, 7.1 Hz, 2H, H-31), 3.20 (*m*, 1H, H-3), 2.86 (*dd*, *J* = 13.9, 4.3 Hz, 1H, H-18), 2.00–1.91 (*m*, 1H, H-16a), 1.90–1.80 (*m*, 2H, H-11a + H-11b), 1.73–1.55 (*m*, 7H, H-22a + H-19a + H-1a + H-15a + H-2a + H-2b + H-16b), 1.54–1.40 (*m*, 4H, H-6a + H-22b + H-9 + H-7a), 1.40–1.24 (*m*, 3H, H-6b + H-21a + H-7b), 1.22 (*t*, *J* = 7.1 Hz, 3H, H-32), 1.20–1.10 (*m*, 2H, H-21b + H-19b), 1.13 (*s*, 3H, H-27), 1.10–1.00 (*m*, 1H, H-15b), 0.98 (*s*, 3H, H-24), 0.98–0.93 (*m*, 1H, H-1b), 0.92 (*s*, 3H, H-30), 0.90 (*s*, 3H, H-25), 0.89 (*s*, 3H, H-29), 0.77 (*s*, 3H, H-23), 0.74 (*s*, 3H, H-26), 0.71 (*m*, 1H, H-5) ppm; ^13^C NMR (125 MHz, CDCl_3_): δ = 177.8 (C-28), 144.0 (C-13), 122.4 (C-12), 79.2 (C-3), 60.2 (C-31), 55.4 (C-5), 47.79 (C-9), 46.7 (C-17), 46.1 (C-19), 41.9 (C-14), 41.5 (C-18), 39.5 (C-8), 38.9 (C-1), 38.6 (C-4), 37.2 (C-10), 34.1 (C-21), 33.3 (C-29), 32.9, (C-7), 32.6 (C-22), 30.8 (C-20), 28.3 (C-24), 27.8 (C-15), 27.4 (C-2), 26.0 (C-27), 23.8 (C-30), 23.6(C-11), 23.2 (C-16), 18.5 (C-6), 17.1 (C-26), 15.7 (C-23), 15.5 (C-25), 14.4 (C-32) ppm; MS (ESI, MeOH): *m*/*z* (%) = 467.3 ([M − H_2_O + H]^+^, 70), 480.2 ([M + H]^+^, 74), 991.5 ([2M + Na]^+^, 100).

### (3β) Methyl 3-{[(1,1-Dimethylethyl)dimethylsilyl]oxy}-urs-12-en-28-oate *(**7**)*

Compound **5** (19.0 g, 0.040 mol) was dissolved in dry DMF (300 mL), and imidazole (7.0 g, 0.103 mol) and TBDMSCl (6.8 g, 0.044 mol) were added. The reaction mixture was stirred for 24 h at 50 °C. The product was precipitated by adding water; it was filtered off and washed with 2 M hydrochloric acid (2 × 20 mL) and water (2 × 50 mL). The resulting solid was dried in a desiccator to yield **7** as a colorless crystalline solid (22.1 g, 95%) [30] m.p. = 153 °C; ${\left[\alpha \right]}_{D}^{20}$ = +57.4° (*c* 0.305, CHCl_3_); R*_f_* = 0.34 (silica gel, hexane/EtOAc, 9:1); IR (KBr): ν = 3446*br*, 2948*m*, 2930*m*, 2856*w*, 1728*w*, 1636*m*, 1460*w*, 1386*w*, 1360*w*, 1252*w*, 1166*w*, 1142*w*, 1108*m*, 1100*m*, 1032*w*, 1004*w*, 836*m* cm^−1^; ^1^H NMR (500 MHz, CDCl_3_): δ = 5.24 (*dd*, *J* = 3.4, 3.4 Hz, 1H, H-12), 3.60 (*s*, 3H, H-32), 3.18 (*dd*, *J* = 11.3, 4.6 Hz, 1H, H-3), 2.22 (*d*, *J* = 11.3 Hz, 1H, H-18), 2.04–1.94 (*m*, 1H, H-16a), 1.93–1.87 (*m*, 2H, H-11a + H-11b), 1.82–1.71 (*m*, 1H, H-2a), 1.70–1.42 (*m*, 10H, H-22a + H-1a + H-21a + H-15a + H-22b + H-1b + H-6a + H-15b + H-7a + H-9), 1.42–1.24 (*m*, 4H, H-19 + H-7b + H-21b + H-6b), 1.07 (*s*, 3H, H-27), 1.06–1.00 (*m*, 2H, H-2b + H-20), 0.94 (*d*, *J* = 6.1 Hz, 3H, H-30), 0.91 (*s*, 3H, H-25), 0.90 (*s*, 3H, H-24), 0.88 (*s*, 9H, H-34), 0.87–0.85 (*m*, 1H, H-16b), 0.86 (*s*, 3H, H-23), 0.85 (*s*, 3H, H-26), 0.74 (*d*, *J* = 6.3 Hz, 3H, H-29), 0.72–0.67 (*m*, 1H, H-5), 0.03 (*s*, 6H, H-31) ppm; ^13^C NMR (125 MHz, CDCl_3_): δ = 178.2 (C-28), 138.3 (C-13), 125.8 (C-12), 79.7 (C-3), 55.5 (C-5), 53.1 (C-18), 51.6 (C-32), 48.3 (C-17), 47.8 (C-9), 42.2 (C-14), 39.7 (C-4), 39.5 (C-8), 39.2 (C-19), 39.0 (C-20), 38.8 (C-1), 37.0 (C-10), 36.8 (C-22), 33.2 (C-7), 30.8 (C-21), 28.7 (C-24), 28.2 (C-2), 27.8 (C-15), 26.1 (C-34), 24.4 (C-16), 23.8 (C-27), 23.5 (C-11), 21.3 (C-30), 18.7 (C-6), 18.3 (C-33), 17.2 (C-29), 17.1 (C-23), 16.3 (C-26), 15.6 (C-25), −3.6 (C-31a), −4.7 (C-31b) ppm; MS (ESI, MeOH): *m*/*z* (%) = 585.2 ([M + H]^+^, 100), 607.4 ([M + Na]^+^, 16), 1192.5 ([2M + Na]^+^, 62).

### (3β) Ethyl 3-{[(1,1-Dimethylethyl)dimethylsilyl]oxy}-olean-12-en-28-oate *(**8**)*

Compound **6** (10.0 g, 0.021 mol) was dissolved in DMF (100 mL), imidazole (3.5 g, 0.520 mol) and TBDMSCl (3.5 g, 0.045 mol) were added. The reaction mixture was stirred for 24 h at 50 °C. The product was precipitated by adding water, which was filtrated off and washed with 2 m hydrochloric acid (2 × 20 mL) and water (2 × 50 mL), and dried in a desiccator to yield **8** as a colorless crystalline solid (11.83 g, 94%) [30]. m.p. = 87 °C; ${\left[\alpha \right]}_{D}^{20}$ = +61.3° (*c* 0.345, CHCl_3_); R*_f_* = 0.75 (silica gel, hexane/EtOAc, 9:1); IR (KBr): ν = 3444*br*, 2950*s*, 2932*s*, 2858*s*, 1636*w*, 1464*m*, 1388*m*, 1362*w*, 1254*m*, 1146*w*, 1102*s*, 1076*m*, 1064*m*, 1050*m*, 1006*m*, 836*m*, 774*m* cm^−1^; ^1^H NMR (500 MHz, CDCl_3_): δ = 5.28 (*dd*, *J* = 3.7, 3.7 Hz, 1H, H-12), 4.16–3.99 (*m*, 2H, H-31), 3.18 (*dd*, *J* = 11.1, 4.6 Hz, 1H, H-3), 2.86 (*dd*, *J* = 14.0, 4.5 Hz, 1H, H-18), 2.02–1.78 (*m*, 3H, H-16a + H-11a + H-11b), 1.77–1.48 (*m*, 9H, H-22a + H-19a + H-2a + H-16b + H-15a + H-1a + H-9 + H-22b + H-6a), 1.48–1.38 (*m*, 2H, H-15b + H-7a), 1.38–1.27 (*m*, 3H, H-6b + H-21b + H-7b), 1.22 (*t*, *J* = 7.1 Hz, 3H, H-32), 1.20–1.14 (*m*, 2H, H-21b + H-19b), 1.13 (*s*, 3H, H-27), 1.10–0.99 (*m*, 1H, H-2b), 0.93–0.89 (*m*, 1H, H-1b), 0.92 (*s*, 3H, H-30), 0.90 (*s*, 3H, H-23), 0.90 (*s*, 3H, H-24), 0.90 (*s*, 3H, H-29), 0.88 (*s*, 9H, H-35), 0.74 (*s*, 3H, H-25), 0.73 (*s*, 3H, H-26), 0.72–0.67 (*m*, 1H, H-5), 0.03 (*s*, 6H, H-33) ppm; ^13^C NMR (125 MHz, CDCl_3_): δ = 177.9 (C-28), 144.0 (C-13), 122.5 (C-12), 79.7 (C-3), 60.2 (C-31), 55.5 (C-5), 47.84 (C-9), 46.7 (C-17), 46.1 (C-19), 41.9 (C-14), 41.5 (C-18), 39.6 (C-4), 39.5 (C-10), 38.6 (C-1), 37.1 (C-8), 34.1 (C-21), 33.3 (C-29), 33.0 (C-7), 32.6 (C-22), 30.9 (C-20), 28.7 (C-24), 27.8 (C-15), 27.8 (C-2), 26.1 (C-32), 26.0 (C-27), 23.8 (C-30), 23.6 (C-11), 23.2 (C-16), 18.7 (C-6), 18.3 (C-34), 17.1 (C-26), 16.3 (C-25), 15.5 (C-23), 14.4 (C-35), −3.6 (C-33b), −4.7 (C-33a) ppm; MS (ASAP): *m*/*z* (%) = 467.2 ([M-Me_2_BuSiOH + H]^+^, 100), 599.3 ([M + H]^+^, 16).

### (3β) 3-{[(1,1-Dimethylethyl)dimethylsilyl]oxy}-urs-12-en-28-ol *(**9**)*

A solution of **7** (20 g, 0.03 mol) dissolved in dry THF (150 mL) was added to a solution of lithium aluminum hydride (5 g, 0.13 mol) in dry THF (300 mL) at 0 °C. The reaction mixture was allowed to warm to room temperature and finally heated for 3 h under reflux. Surplus lithium aluminum hydride was deactivated by adding THF/water (1:1) at 0 °C. An aqueous solution of sodium hydroxide (20 mL) was added, and the mixture was stirred for 15 min. The precipitate was filtered off and washed with diethyl ether (2 × 100 mL). The aqueous phase was extracted with diethyl ether (2 × 50 mL) and the combined organic extracts were washed with brine and dried over magnesium sulfate. After filtration, the solvent was removed under reduced pressure to yield crude **9.** Further purification with column chromatography afforded (silica gel, hexane/EtOAc 9:1) **9** as a colorless crystalline solid (18.2 g, 96%). m.p. = 113–116 °C; ${\left[\alpha \right]}_{D}^{20}$ = +59.8° (*c* 0.325, CHCl_3_); R*_f_* = 0.25 (silica gel, hexane/EtOAc, 9:1); IR (KBr): 3448*br*, 2929*s*, 2856*s*, 1636*w*, 1461*s*, 1388*m*, 1254*m*, 1101*s*, 1069*s*, 1003*m*, 883*m*, 836*s*, 774*m* cm^−1^; ^1^H NMR (500 MHz, CDCl_3_): δ = 5.13 (*dd*, *J* = 3.4, 3.4 Hz, 1H, H-12), 3.52 (*d*, *J* = 10.9 Hz, 1H, H-28a), 3.22–3.16 (*m*, 2H, H-28b + H-3), 1.98–1.86 (*m*, 3H, H-16a + H-11a + H-11b), 1.83–1.72 (*m*, 1H, H-15a), 1.65–1.44 (*m*, 8H, H-2a + H-1a + H-22a + H-7a + H-6a + H-9 + H-21a + H-2b), 1.44–1.30 (*m*, 5H, H-19 + H-22b + H-6b + H-18 + H-7b), 1.28–1.15 (*m*, 2H, H-16b + H-21b), 1.10 (*s*, 3H, H-27), 0.98 (*s*, 3H, H-26), 1.03–0.93 (*m*, 2H, H-15b + H-1b), 0.94 (*s*, 3H, H-25), 0.94 (*d*, *J* = 6.7 Hz, 3H, H-30), 0.91 (*s*, 3H, H-24), 0.89 (*s*, 9H, H-33), 0.89 (*m*, 1H, H-20), 0.81 (*d*, *J* = 5.6 Hz, 3H, H-29), 0.75 (*s*, 3H, H-23), 0.74–0.67 (*m*, 1H, H-5), 0.03 (*s*, 6H, H-31) ppm; ^13^C NMR (125 MHz, CDCl_3_): δ = 138.8 (C-13), 125.3 (C-12), 79.6 (C-3), 70.1 (C-28), 55.4 (C-5), 54.2 (C-18), 47.9 (C-9), 42.2 (C-14), 40.2 (C-8), 39.6 (C-19), 39.5 (C-20), 39.5 (C-4), 39.0 (C-1), 38.2 (C-17), 36.9 (C-10), 35.3 (C-22), 33.1 (C-7), 30.8 (C-21), 28.7 (C-24), 27.8 (C-2), 26.2 (C-15), 26.1 (C-33), 23.6 (C-11), 23.5 (C-16), 23.5 (C-27), 21.5 (C-30), 18.7 (C-6), 18.3 (C-32), 17.5 (C-29), 16.9 (C-26), 16.3 (C-23), 15.9 (C-25), −3.6 (C-31a), −4.7 (C-31b) ppm; MS (ASAP): *m*/*z* (%) = 425.6 ([M-Me_2_BuSiOH + H]^+^, 100), 557.8 ([M + H]^+^, 20).

### (3β) 3-{[(1,1-Dimethylethyl)dimethylsilyl]oxy}-olean-12-en-28-ol *(**10**)*

A solution of **8** (10.0 g, 0.017 mol) in dry THF (100 mL) was added slowly to a solution of lithium aluminum hydride (3.0 g, 0.080 mol) in dry THF (150 mL) at 0 °C. The solution was heated under reflux until TLC showed the reaction to be complete. The mixture was cooled to 0 °C and surplus of LiAlH_4_ was deactivated by adding THF/water (1:1). An aqueous solution (2 M) of sodium hydroxide (10 mL) was added, and the mixture was stirred for 15 min. The solid was filtered off, washed with diethyl ether (2 × 50 mL), and the organic phase was washed with brine (25 mL). The aqueous phase was washed with diethyl ether (2 × 50 mL). The combined organic phases were dried over magnesium sulfate and concentrated under reduced pressure. After column chromatography (silica gel, hexane/EtOAc 9:1) compound **10** was obtained as a colorless crystalline solid (8.7 g, 95%). m.p. = 106–108 °C (lit.: 110–111 °C) [15]; ${\left[\alpha \right]}_{D}^{20}$ = +64.3° (*c* 0.345, CHCl_3_) (lit.: +61.0° (*c* 0.5, CHCl_3_)) [15]; R*_f_* = 0.28 (silica gel, hexane/EtOAc, 9:1); IR (KBr): 3444*br*, 2950*s*, 2932*s*, 2858*s*, 1636*w*, 1464*m*, 1388*m*, 1362*w*, 1254*m*, 1102*s*, 1076*m*, 1064*m*, 1050*m*, 1006*m*, 836*m*, 774*m* cm^−1^; ^1^H NMR (400 MHz, CDCl_3_): δ = 5.19 (*dd*, *J* = 3.6, 3.6 Hz, 1H, H-12), 3.55 (*d*, *J* = 10.5 Hz, 1H, H-28a), 3.22 (*d*, *J* = 10.6 Hz, 1H, H-28b), 3.21–3.16 (*m*, 1H, H-3), 2.06–1.95 (*m*, 1 H, H-18), 1.94–1.77 (*m*, 3 H, H-16a + H-11a + H-11b), 1.78–1.65 (*m*, 2H, H-19a + H-2a), 1.65–1.24 (*m*, 11H, H-15a + H-1a + H-6a + H-7a + H-9 + H-22a + H-15b + H-6b + H-7b + H-22b + H-21a), 1.24–1.12 (*m*, 2H, H-16b + H-21b), 1.16 (*s*, 3H, H-27), 1.12–0.96 (*m*, 2H, H-19b + H-2b), 0.95–0.87 (*m*, 1H, H-1b), 0.93 (*s*, 3H, H-26), 0.93 (*s*, 3H, H-23), 0.91 (*s*, 3H, H-30), 0.89 (*s*, 9H, H-33), 0.89 (*s*, 3H, H-24), 0.89 (*s*, 3H, H-29), 0.75 (*s*, 3H, H-25), 0.71 (*m*, 1H, H-5), 0.03 (*s*, 6H, H-31) ppm; ^13^C NMR (100 MHz, CDCl_3_): δ = 144.3 (C-13), 122.6 (C-12), 79.6 (C-3), 69.9 (C-28), 55.4 (C-5), 47.8 (C-9), 46.6 (C-19), 42.5 (C-18), 41.9 (C-14), 40.0 (C-8), 39.5 (C-4), 38.8 (C-1), 37.1 (C-17), 37.0 (C-10), 34.3 (C-21), 33.4 (C-29), 32.8 (C-22), 31.2 (C-20), 31.1 (C-7), 28.7 (C-30), 27.8 (C-15), 26.1 (C-33), 26.1 (C-24), 25.7 (C-2), 23.8 (C-27), 23.7 (C-11), 22.2 (C-16), 18.7 (C-6), 18.3 (C-32), 16.9 (C-26), 16.3 (C-25), 15.7 (C-23), −3.6 (C-31a), −4.7 (C-31b) ppm; MS (ASAP): *m*/*z* (%) = 425.5 ([M − Me_2_BuSiOH + H]^+^, 100), 557.6 ([M + H]^+^, 24).

### (3β) 3-{[(1,1-Dimethylethyl)dimethylsilyl]oxy}-28-{[(trifluoromethyl)sulfonyl]oxy}-urs-12-en *(**11**)*

Compound **9** (1.0 g, 1.8 mmol) was dissolved in DCM (50 mL). At 0 °C, pyridine (0.3 mL, 3.71 mmol) and trifluoromethanesulfonic anhydride (0.45 mL, 2.7 mmol) were added slowly. The reaction mixture was stirred for 15 min at 0 °C. Then NaHCO_3_ (20 mL, aq, satd.) was added, and the mixture was washed with 2 M hydrochloric acid. The organic phase was dried over magnesium sulfate, filtered, and the solvent was removed under reduced pressure to yield **11** as a colorless amorphous solid (1.13 g, 91%). R*_f_* = 0.46 (silica gel, hexane) ^1^H NMR (500 MHz, CDCl_3_): δ = 5.17 (*dd*, *J* = 3.5, 3.5 Hz, 1H, H-12), 4.19 (*d*, *J* = 9.2 Hz, 1H, H-28a), 3.72 (*d*, *J* = 9.3 Hz, 1H, H-28b), 3.18 (*dd*, *J* = 11.2, 4.6 Hz, 1H, H-3), 2.06–1.94 (*m*, 1H, H-16a), 1.94–1.89 (*m*, 2H, H-11a + H-11b), 1.89–1.69 (*m*, 1H, H-15a), 1.69–1.62 (*m*, 1H, H-22a), 1.63–1.31 (*m*, 7H, H-2a + H-1a + H-7a + H-6a + H-9 + H-21a + H-2b), 1.31–1.15 (*m*, 7H, H-19 + H-18 + H-22b + H-6b + H-7b + H16b + H-21b), 1.11 (*s*, 3H, H-27), 1.09–1.02 (*m*, 1H, H-15b), 1.00 (*s*, 3H, H-26), 0.99–0.94 (*m*, 1H, H-1b), 0.94 (*s*, 3H, H-23), 0.94 (*s*, 3H, H-30), 0.91 (*s*, 3H, H-24), 0.88 (*s*, 9H, H-33), 0.88 (*m*, 1H, H-20), 0.81 (*d*, *J* = 5.4 Hz, 3H, H-29), 0.75 (*s*, 3H, H-25), 0.73–0.68 (*m*, 1H, H-5), 0.03 (*s*, 6H, H-31) ppm; ^13^C NMR (125 MHz, CDCl_3_): δ = 137.2 (C-13), 127.0 (C-12), 118.8 (*q*, *J* = 319.8 Hz, C-34), 84.3 (C-3), 79.6 (C-28), 55.4 (C-5), 53.7 (C-18), 47.8 (C-9), 42.1 (C-14), 40.2 (C-8), 39.5 (C-4), 39.4 (C-19), 39.3 (C-20), 39.0 (C-1), 37.9 (C-17), 36.9 (C-10), 35.2 (C-22), 32.9 (C-7), 30.2 (C-21), 28.7 (C-24), 27.8 (C-2), 26.1 (C-33), 25.8 (C-15), 23.6 (C-11), 23.5 (C-27), 23.0 (C-16), 21.3 (C-30), 18.6 (C-6), 18.3 (C-32), 17.2 (C-29), 16.6 (C-26), 16.3 (C-23), 15.9 (C-25), −3.6 (C-31a), −4.7 (C-31b) ppm; MS (ASAP): *m*/*z* (%) = 557.5.2 ([M-Me_2_BuSiOH + H]^+^, 14), 407.5 ([M-HOTf-Me_2_BuSiOH + H]^+^, 100).

### (3β) 3-{[(1,1-Dimethylethyl)dimethylsilyl]oxy}-olean-12-en-28-methansulfonate *(**12**)*

Compound **10** (5 g, 9.0 mmol) was dissolved in DCM (200 mL). At 0 °C triethylamine (4 mL, 28.7 mmol) and methanesulfonyl chloride (0.76 mL, 9.87 mmol) were added. The reaction mixture was stirred for an hour at room temperature and quenched by adding water (50 mL) and 2 M hydrochloric acid (20 mL). The organic phase was separated. The aqueous phase was extracted with DCM (50 mL). The combined organic extracts were washed with brine and dried over magnesium sulfate. Purification by column chromatography (silica gel, hexane/EtOAc, 9:1) afforded **12** as a colorless crystalline solid (5.31 g, 91%). m.p. = 71–74 °C; ${\left[\alpha \right]}_{D}^{20}$ = +66.4° (*c* 0.320, CHCl_3_); R*_f_* = 0.25 (silica gel, hexane/EtOAc, 9:1); IR (KBr): 3444*br*, 2952*s*, 2930*s*, 2856*s*, 1638*w*, 1460*m*, 1386*m*, 1358*s*, 1254*m*, 1176*s*, 1100*m*, 1072*m*, 1004*m*, 954*s*, 838*s*, 776*m*, 530*m* cm^−1^; ^1^H NMR (500 MHz, CDCl_3_): δ = 5.23 (*dd*, *J* = 3.5, 3.5 Hz, 1H, H-12), 4.18 (*d*, *J* = 9.3 Hz, 1H, H-28a), 3.78 (*d*, *J* = 9.3 Hz, 1H, H-28b), 3.18 (*dd*, *J* = 11.1, 4.5 Hz, 1H, H-3), 2.98 (*s*, 3H, H-34), 2.06–1.84 (*m*, 3H, H-16a + H-11a + H-11b), 1.85–1.74 (*m*, 1H, H-2a), 1.70–1.43 (*m*, 11H, H-1a + H-15a + H-19a + H-21a + H-6a + H-15b + H-7a + H-22a + H-9+ H-21b + H-6b), 1.42–1.33 (*m*, 4H, H-16a + H-19b + H-18 + H-22b), 1.33– 1.18 (*m*, 2H, H-16b + H-7b), 1.17 (*s*, 3H, H-27), 1.13–1.07 (*m*, 1H, H-2b), 1.07–0.99 (*m*, 1H, H-1b), 0.96 (*s*, 3H, H-26), 0.92 (*s*, 3H, H-24), 0.91 (*s*, 3H, H-23), 0.90 (*s*, 3H, H-30), 0.89 (*s*, 9H, H-33), 0.88 (*s*, 3H, H-29), 0.75 (*s*, 3H, H-25), 0.73–0.67 (*m*, 1H, H-5), 0.03 (*s*, 6H, H-32) ppm; ^13^C NMR (125 MHz, CDCl_3_): δ = 143.2 (C-13), 123.7 (C-12), 79.6 (C-3), 76.7 (C-28), 55.4 (C-5), 47.7 (C-9), 46.2 (C-19), 42.4 (C-18), 41.8 (C-14), 40.0 (C-8), 39.5 (C-4), 38.8 (C-1), 37.1 (C-34), 37.0 (C-17), 36.5 (C-10), 33.9 (C-21), 33.2 (C-29), 32.7 (C-22), 31.4 (C-20), 31.0 (C-7), 28.7 (C-30), 27.8 (C-15), 26.2 (C-24), 26.1 (C-33), 25.5 (C-2), 23.8 (C-11), 23.7 (C-27), 21.9 (C-16), 18.6 (C-6), 18.3 (C-32), 16.8 (C-26), 16.3 (C-25), 15.7 (C-23), −3.6 (C-31a), −4.7 (C-31b) ppm; MS (ASAP): *m*/*z* (%) = 407.3 ([M-Me_2_BuSiOH-MeSO_3_H + H]^+^, 10), 503.3 ([M − Me_2_BuSiOH + H]^+^, 60).

### (3β) 3-{[(1,1-Dimethylethyl)dimethylsilyl]oxy}-urs-12-en *(**13**)*

A solution of **11** (1.0 g, 1.45 mmol) in dry THF (10 mL) was added at 0 °C to a solution of lithium aluminum hydride (0.2 g, 5.27 mmol) in dry THF (20 mL). The reaction mixture was stirred for four hours at room temperature. Work-up as described above, followed by column chromatography (silica gel, hexane), gave **13** as a colorless crystalline solid (0.68 g, 86%). m.p. = 179–182 °C; ${\left[\alpha \right]}_{D}^{20}$ = +69.4° (*c* 0.305, CHCl_3_); R*_f_* = 0.92 (silica gel, hexane); IR (KBr): 3448*br*, 2926*s*, 2855*s*, 1636*w*, 1458*m*, 1386*m*, 1253*m*, 1101*s*, 1069*m*, 1004*w*, 835*m*, 774*m* cm^−1^; ^1^H NMR (500 MHz, CDCl_3_): δ = 5.13 (*dd*, *J* = 3.6, 3.6 Hz, 1H, H-12), 3.20 (*dd*, *J* = 11.2, 4.6 Hz, 1H, H-3), 2.05–1.95 (*m*, 1H, H-16a), 1.95–1.79 (*m*, 3H, H-11a + H-11b + H-15a), 1.66–1.58 (*m*, 2H, H-2a + H-1a), 1.58–1.21 (*m*, 12H, H-6a + H-7a + H-9 + H-2b + H-22a + H-6b + H-21a + H-7b + H-18 + H-19 + H-22b + H-21b), 1.07 (*s*, 3H, H-27), 1.01 (*s*, 3H, H-26), 1.00–0.96 (*m*, 2H, H-15b + H-1b), 0.95 (*s*, 3H, H-23), 0.92 (*d*, *J* = 6.0 Hz, 3H, H-30), 0.91 (*s*, 3H, H-24), 0.89 (*s*, 9H, H-33), 0.89–0.83 (*m*, 2H, H-20 + H-16b), 0.80 (*s*, 3H, H-28), 0.79 (*d*, *J* = 6.0 Hz, 3H, H-29), 0.76 (*s*, 3H, H-25), 0.71 (*m*, 1H, H-5), 0.04 (*s*, 6H, H-31) ppm; ^13^C NMR (125 MHz, CDCl_3_): δ = 139.7 (C-13), 124.7 (C-12), 79.7 (C-3), 59.3 (C-18), 55.5 (C-5), 48.0 (C-9), 42.3 (C-14), 41.7 (C-22), 40.2 (C-8), 39.8 (C-19), 39.8 (C-20), 39.5 (C-4), 39.0 (C-1), 37.0 (C-10), 33.9 (C-17), 33.2 (C-7), 31.5 (C-21), 28.9 (C-28), 28.7 (C-24), 28.3 (C-16), 27.9 (C-2), 26.8 (C-15), 26.1 (C-33), 23.6 (C-11), 23.4 (C-27), 21.6 (C-30), 18.7 (C-6), 18.3 (C-32), 17.6 (C-29), 17.1 (C-26), 16.3 (C-25), 15.9 (C-23), −3.6 (C-32a), −4.7 (C-32b) ppm; MS (ASAP): *m*/*z* (%) = 409.2 ([M − Me_2_BuSiOH + H]^+^, 100), 541.3 ([M + H]^+^, 2).

### (3β) 3-{[(1,1-Dimethylethyl)dimethylsilyl]oxy}-olean-12-en *(**14**)*

Reduction of **12** (4 g, 6.3 mmol) in THF (50 mL) at 0 °C with lithium aluminum hydride (1.2 g, 31.6 mmol) in THF (100 mL), as described above, followed by column chromatography (silica gel, hexane) gave **14** as a colorless crystalline solid (3.14 g, 92%). m.p. = 174–176 °C (lit.: 179.5–180.5 °C) [15]; ${\left[\alpha \right]}_{D}^{20}$ = +72.6° (*c* 0.315, CHCl_3_) (lit.: +78.6° (*c* 0.21, CHCl_3_)) [15]; R*_f_* = 0.94 (silica gel, hexane); IR (KBr): 3447*br*, 2949*s*, 2930*s*, 2856*s*, 1636*w*, 1461*m*, 1387*m*, 1253*m*, 1100*m*, 1076*m*, 884*m*, 835*s*, 771*m* cm^−1^; ^1^H NMR (500 MHz, CDCl_3_): δ = 5.18 (*dd*, *J* = 3.5, 3.5 Hz, 1H, H-12), 3.19 (*dd*, *J* = 11.1, 4.6 Hz, 1H, H-3), 2.04–1.91 (*m*, 2H, H-16a + H-18), 1.93–1.72 (*m*, 3H, H-11a + H-11b + H-15a), 1.72–1.37 (*m*, 9H, H-19a + H-2a + H-1a + H-6a + H-9 + H-7a + H-2b + H-22a + H-6b), 1.37–1.28 (*m*, 2H, H-21a + H-7b), 1.29–1.17 (*m*, 1H, H-22b), 1.13 (*s*, 3H, H-27), 1.13–1.07 (*m*, 1H, H-21b), 1.06–0.99 (*m*, 1H, H-19b), 0.99–0.95 (*m*, 1H, H-15b), 0.96 (*s*, 3H, H-26), 0.94–0.90 (*m*, 1H, H-1b), 0.94 (*s*, 3H, H-25), 0.91 (*s*, 3H, H-24), 0.89 (*s*, 9H, H-33), 0.88 (*s*, 3H, H-30), 0.87 (*s*, 3H, H-29), 0.84 (*s*, 3H, H-28), 0.83–0.77 (*m*, 1H, H-16b), 0.76 (*s*, 3H, H-23), 0.74–0.69 (*m*, 1H, H-5), 0.04 (*s*, 6H, H-31) ppm; ^13^C NMR (125 MHz, CDCl_3_): δ = 145.3 (C-13), 122.0 (C-12), 79.7 (C-3), 55.5 (C-5), 47.9 (C-9), 47.4 (C-18), 47.0 (C-19), 41.9 (C-14), 40.00 (C-8), 39.5 (C-4), 38.8 (C-1), 37.3 (C-22), 37.0 (C-10), 34.9 (C-21), 33.5 (C-30), 32.9 (C-7), 32.7 (C-17), 31.3 (C-20), 28.7 (C-24), 28.6 (C-28), 27.8 (C-2), 27.1 (C-16), 26.3 (C-15), 26.2 (C-27), 26.1 (C-33), 23.9 (C-29), 23.7 (C-11), 18.8 (C-6), 18.3 (C-32), 17.0 (C-26), 16.3 (C-23), 15.7 (C-25), −3.6 (C-31a), −4.7 (C-31b) ppm; MS (ASAP): *m*/*z* (%) = 409.2 ([M-Me_2_BuSiOH + H]^+^, 100), 541.3 ([M + H]^+^, 18).

## 4. Materials and Methods

Melting points are uncorrected (Leica hot stage microscope), NMR spectra were recorded using the Varian spectrometers Gemini 2000 or Unity 500 (δ given in ppm, *J* in Hz, internal Me_4_Si; typical experiments: HH-COSY, gHMBC, gHMQC, NOESY, DQFCOSY), ESI-MS spectra were taken on a Finnigan MAT LCQ 7000 (electrospray, voltage 4.1 kV, sheath gas nitrogen) instrument. ASAP-MS spectra were taken on an Advion expression CMS-L with an ASAP/APCI Ion source (capillary voltage 150 V, capillary temperature 220 °C, and voltage of the ion source: 15 V; APCI source temperature 300 °C with 5 µA). The optical rotation was measured on a PerkinElmer polarimeter at 20 °C; TLC was performed on silica gel (Merck 5554); the solvents were dried according to usual procedures. Oleanolic and ursolic acid were obtained from “betulinines” (Stribrna Skalica, Czech Republic) in bulk quantities.
